# Estrogen Receptor Silencing Induces Epithelial to Mesenchymal Transition in Human Breast Cancer Cells

**DOI:** 10.1371/journal.pone.0020610

**Published:** 2011-06-21

**Authors:** Sanaa Al Saleh, Fahd Al Mulla, Yunus A. Luqmani

**Affiliations:** 1 Faculty of Pharmacy, Kuwait University, Safat, Kuwait; 2 College of Graduate Studies, Kuwait University, Safat, Kuwait; 3 Faculty of Medicine, Kuwait University, Safat, Kuwait; Beth Israel Deaconess Medical Center, United States of America

## Abstract

We propose the hypothesis that loss of estrogen receptor function which leads to endocrine resistance in breast cancer, also results in trans-differentiation from an epithelial to a mesenchymal phenotype that is responsible for increased aggressiveness and metastatic propensity. siRNA mediated silencing of the estrogen receptor in MCF7 breast cancer cells resulted in estrogen/tamoxifen resistant cells (pII) with altered morphology, increased motility with rearrangement and switch from a keratin/actin to a vimentin based cytoskeleton, and ability to invade simulated components of the extracellular matrix. Phenotypic profiling using an Affymetrix Human Genome U133 plus 2.0 GeneChip indicated geometric fold changes ≥3 in approximately 2500 identifiable unique sequences, with about 1270 of these being up-regulated in pII cells. Changes were associated with genes whose products are involved in cell motility, loss of cellular adhesion and interaction with the extracellular matrix. Selective analysis of the data also showed a shift from luminal to basal cell markers and increased expression of a wide spectrum of genes normally associated with mesenchymal characteristics, with consequent loss of epithelial specific markers. Over-expression of several peptide growth factors and their receptors are indicative of an increased contribution to the higher proliferative rates of pII cells as well as aiding their potential for metastatic activity. Signalling molecules that have been identified as key transcriptional drivers of epithelial to mesenchymal transition were also found to be elevated in pII cells. These data support our hypothesis that induced loss of estrogen receptor in previously estrogen/antiestrogen sensitive cells is a trigger for the concomitant loss of endocrine dependence and onset of a series of possibly parallel events that changes the cell from an epithelial to a mesenchymal type. Inhibition of this transition through targeting of specific mediators may offer a useful supplementary strategy to circumvent the effects of loss of endocrine sensitivity.

## Introduction

Frequent and unpredictable onset of an endocrine drug resistant phenotype continues to pose significant problems for long term management of breast cancer patients [1], [2]. Variously derived models of antiestrogen resistant cells have failed to highlight any single mechanism entirely responsible for this phenomenon [3]. Over-expression of tyrosine kinase receptors (RTKs) for a variety of other cellular mediators of proliferation has been frequently reported [3]–[5]. Convergence at downstream elements such as MAPK, pI3K, SRC and AKT (all gene symbols used in this report follow approved nomenclature as defined in the HUGO database; http://www.genenames.org/index.html) has been implicated in situations where the classical route via genomic estrogen response elements has become redundant. Bi-directional crosstalk between ER and RTK mediated signalling is implicated as a major route to endocrine resistance [6] with identification of several potential contributing factors including GATA3, GATA4, FOXp3, pAX2, NCOA3 and SRC members [7]–[9]. Endocrine resistance is generally characterised by accelerated growth and increased aggressive behaviour, and associated with morphological changes characteristic of cells undergoing epithelial-to-mesenchymal transition (EMT) [10]. Epithelial carcinoma cells may acquire a mesenchymal-like state to facilitate migration and invasion and undergo reversion (mesenchymal to epithelial transition; MET) to form organized tumourigenic nodules at lodgement sites. During EMT and MET, a bimodal communication exists between the host fibroblasts, extracellular matrix/basement membrane and the immune cells that involves a series of transcriptional re-programming steps requiring participation of several transcription factors including ZEB1/TCF8, SNAIL1, ZEB2, SNAIL2, E12/E47, FOXC2, GOOSECOID, and TWIST [11], [12]. A defining feature of EMT is the loss of the homotypic cell adhesion molecule E-cadherin (CDH1) and the occludins [13], [14] which together with claudins and tight junction proteins are integral components of adherens junctions forming the cohesive architecture of normal epithelia. This is very likely an initiating step for transition of breast tumours from a benign to an invasive state [15]–[17], leading to vascular metastasis [18]–[20]. Expression of the E-cadherin repressor proteins, SNAIL, TWIST, ZEB1 (EF1), LCN2 and KLF8 is increased in EMT, with concurrent loss of cytokeratins such as CK8, 18, and 19 and the appearance of N-cadherin (CDH12) and/or cadherin-11 [13], [14], [21] and most critically of vimentin (VIM), the archetypal mesenchymal marker that is over-expressed in breast carcinomas undergoing EMT and exhibiting increased cell migration and invasion [10], [22]. It is co-ordinately regulated together with other mesenchymal markers, such as the extracellular matrix molecule tenascin C [23], whose expression in human breast carcinomas correlates positively with ERBB2 over-expression and down-regulation of ER. Vimentin, along with fibronectin (FN1), is up-regulated by LCN2 [24] in contrast to E-cadherin and cytokeratins, Another EMT marker is AXL, which is a member of the TAM (TYRO-AXL-MER) receptor tyrosine kinases that share the vitamin K-dependent ligand growth arrest-specific gene 6 [21]. TAM family RTKs regulate a diverse range of cellular responses including cell survival, proliferation, autophagy, migration, angiogenesis, platelet aggregation, and natural killer cell differentiation [25]. HOX genes are also associated with tumour progression and have been implicated in many types of cancers including lung, prostate, ovarian, and endometrial cancer. Over-expression of HOXB7 in SKBR3 breast cancer cells was found to directly or indirectly regulate the expression of many angiogenic and growth factors, including bFGF, VEGF, interleukin 8, ANG1 and 2, and matrix metalloproteinase (MMP) 9, and resulted in formation of well-vascularized tumours when grown as xenografts in nude mice [26], [27]. HOXB9 has a similar effect leading to increased cell motility and acquisition of mesenchymal phenotypes [28]. Another mesenchymal/EMT marker is FSP1/S100A4 that has provided *in vivo* evidence implicating EMT in onset of metastasis [13], facilitated through activation of MMP9 and SPARC [14]. A family of more than 28 members, MMPs are up-regulated in nearly every tumour type and are intimately involved in cancer progression through cleavage and release of bioactive molecules that inhibit apoptosis and stimulate invasion, as well as through degradation of extracellular matrix (ECM) components that promote tumour cell growth [29].

In a recently established cellular model of endocrine resistance [30], we provided preliminary evidence suggesting that loss of ER function can result in trans-differentiation of the epithelial phenotype. There is already sporadic evidence, in the literature, of the association of some characteristics of EMT with ER status, particularly in the case of vimentin [31]. Several studies have examined gene expression profiles of ER+ve and ER-ve breast cancers [32] and performed microarray analyses on breast tissues in an attempt to sub-classify patients on the basis of their global pattern of gene expression [33]–[36]. Recently, expression profiling has been used to demonstrate a link between drug resistance and the adoption of an EMT phenotype in MCF7 cells [37]. Surprisingly however, phenotypic and transcriptome alterations in breast cancer cells secondary to ER silencing have hardly been explored. In this report we present data to show that induced loss of ER directly results in the development of EMT and provides a link between this process and endocrine resistance.

## Methods

### Cell lines

MCF7 and MDA231 human breast carcinoma cell lines, derived from the ATCC (American Type Culture Collection, VA, USA) were maintained at 37°C in a humidified atmosphere of 5% CO_2_ in DMEM supplemented with 10% foetal bovine serum (FBS), 600 µg/ml L-glutamine, 100 U/ml penicillin, 100 µg/ml streptomycin and 6 ml/500 ml 100 x non-essential amino acids (Invitrogen, CA, USA). Endocrine independent cell line pII was established by transfection of MCF7 with ER directed shRNA plasmid as described previously [30], [38]. Another transfected cell line designated E2 was established that had retained sensitivity to estrogen and tamoxifen (presumably as a result of failure to produce siRNA) and was used as an additional control.

### Transfections

Complementary oligos (synthesised by Eurogentec, Belgium) containing an ER directed shRNA sequence 5′GCTTCAGGCTACCATTATGttcaagagacataATGGTAGCCTGAAGCttttttacgcgt-3′ (ER sense and anti-sense shown in uppercase and intervening loop sequence and transcription termination sequences shown in lower case) were hybridised, purified and cloned into the Hind III/ Xho1 site of pSingle-tTS vector following the manufacturer's cloning protocol (Clontech, UK). Standard CaCl_2_ transformation into DH5α E coli was followed by plasmid isolation and purification using Qiagen mini kits (Qiagen, CA, USA). Transfection into MCF7 was performed using TransFast reagent (Promega, Southampton, UK) as per the manufacturer's protocol. Cell lines SY1.2 and SY2.5 were established from two G418 (1 mg/ml) resistant clones.

### Cell proliferation

Various numbers of cells from 10^4^–6.10^4^ were seeded into triplicate wells of 12-well plates and growth assessed over 4–6 days, following trypsinisation and resuspension in medium, by direct cell counting using a haematocytometer.

### Cell motility assay

Cells were grown to 80–90% confluency in 12 well plates. A scratch was made with a yellow eppendorf tip through the center of the monolayer. Detached cells were removed by gentle washing with PBS and fresh medium added. The width of the scratch was monitored by light microscopy after 24 and 48 h to determine the extent if any of cell migration into the scratched space.

### Phalloidin staining

Exponentially growing cells were washed with PBS, fixed for 5 min in 3.7% formaldehyde/PBS, washed in PBS, dehydrated with acetone and permeabilised with 0.1% Triton X-100 in PBS. After a further wash with PBS, cells were stained with phalloidin conjugate solution (50 mg/ml) in PBS containing 1% DMSO for 40 min, at room temperature. Unbound conjugate was removed by several washes with PBS before histological visualisation and photography using LSM 510 META confocal microscope (Carl Zeiss, Germany).

### Invasion through basement membrane extract

This assay was performed using 24-well cell invasion chambers from Trevigen (MD, USA) according to the manufacturer's instructions. Plates were equilibrated for 1 h at room temperature. Inserts were rehydrated by addition of 100 µl of warm (37°C) 1 x Rehydration Solution and incubated at 37°C for 1 h. Trypsinised pelleted cells were rinsed with wash buffer and re-suspended at 10^6^ cells/ml in serum free medium. Cells (100 µl) were added to the top chamber of each well. After incubation, the top chambers were aspirated and washed with 100 µl of warm wash buffer. The bottom chambers were also aspirated and washed twice with 500 µl wash buffer. Calcein AM solution (500 µl) was added to the bottom chambers and the unit re-assembled, followed by incubation at 37°C for 1 h. Chambers were disassembled and the plates read in a Fluroskan Ascent plate reader (Thermo-Electron Corporation, USA) at 520 nm emission with 485 nm excitation The number of cells migrating through the matrix was determined using a standard curve constructed with known numbers of pII cells.

### Agarose spot invasion assay

One or more agarose spots were made by pipetting 10 µl of sterile 0.5% molten low melting agarose (prepared in isotonic PBS) into wells of a 12-well plate and allowing to solidify for several minutes at 4°C. About 10^4^–10^5^ cells re-suspended in 1 ml DMEM were then pipetted into each well and left at 37°C for 24–48 h. During this time plates were periodically examined to monitor the penetration of cells into the agarose spot.

### Realtime quantitative PCR

Total cellular RNA was extracted from frozen cell pellets using the RNeasy kit (Qiagen, USA) according to the manufacturer's protocol, and 2 µg (in 20 µl) converted to cDNA using a high capacity cDNA reverse transcriptase kit (Applied Biosystems, CA, USA). Quantitative realtime PCR was performed on 1 µl cDNA using a standard multiplexed TaqMan PCR kit protocol [manufacturer proprietary primer/probe mixes: FAM/TAMRA labelled target was combined with Joe labelled normaliser actin gene] to determine expression of ER, keratin 19, E-cadherin, N-cadherin, fibronectin, vimentin, plasminogen activator-urokinase (PLAU) and CD68 and actin. The 20 µl reactions were performed in a 96-well plate on an Applied Biosystems 7500HT Sequence Detection System by incubation at 95°C for 10 min, followed by 40 cycles of 95°C for 15 s and 60°C for 1 min. The raw threshold cycle (CT) values were analysed by the ΔΔCt method using the spreadsheet developed by Pfaffl [39] to determine normalized expression ratios of target genes.

### Microarray analysis

High quality RNA prepared from MCF7 and pII cells with array grade RNA extraction kit from SABiosciences (MD, USA) was converted to cDNA using the Affymetrix One-Cycle cDNA Synthesis Kit (Affymetrix Technologies, CA, USA). *In vitro* transcription and hybridization in triplicate for each cell line onto Affymetrix Human Genome U133 plus 2.0 GeneChips were performed as previously described [40]. All samples passed the Affymetrix quality control algorithm. The Cel files from each sample were exported to Partek Genomics Suite V 6.5 for RMA background correction, quantile normalisation with GC content adjustment (Partek Incorporated, St Louis, Missouri). ANOVA *p* value for differentially expressed genes was set to 0.05 or less. Gene Ontology (GO) Enrichment Analysis and Visualization tool (Gorilla) was used to determine Gene Ontology categories (http://cbl-gorilla.cs.technion.ac.il/). Functional analysis was performed on the entire data set with the Ingenuity Pathways Analysis (IPA) package (Ingenuity Systems; http://www.ingenuity.com). This programme works by mapping the average gene expression of transcripts to hypothetical networks available in the Ingenuity database and ranks them by a score that indicates the likelihood of the genes in a network being found together due to random chance. In addition, the data was mined for genes with specific functional relevance to endocrine function, metastasis and EMT by manual selection. All the Affymetrix raw data is MIAME compliant and has been deposited in a MIAME compliant database GEO, as detailed on the website: http://www.ncbi.nlm.nih.gov/geo/query/acc.cgi?acc=GSE27473.

### Statistical analyses

Means of experimental groups were compared with those of other experimental groups or controls using the student's t-test. Statistical significance was assumed at *p* values ≤0.05.

## Results

### Cell morphology and proliferation

**Figure 1:**
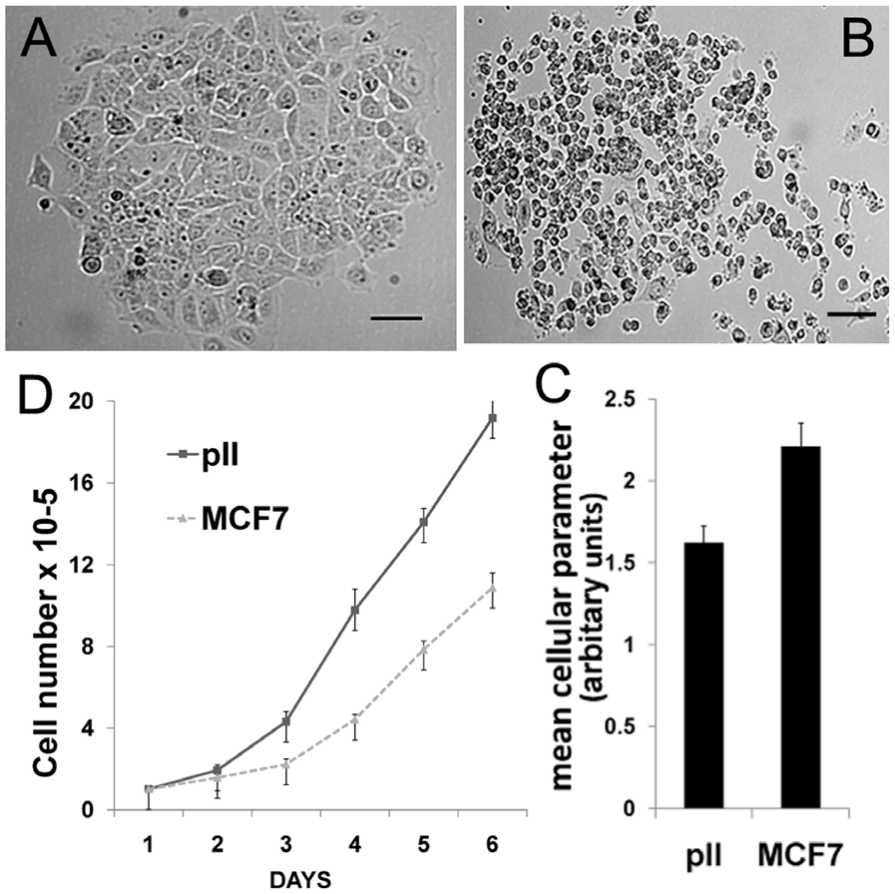
Morphology and growth rates of MCF7 and pII cells. Panels A and B: Phase-contrast optical photomicrographs of single colonies of MCF7 and pII respectively. Scale bar represents 100 µm. Panel C: Cell shape analysis was performed using an ImageJ freeware package; histograms show mean cell parameter ± SEM of 4 independent determinations quantifying morphological differences between MCF7 and pII cells (p < 0.04). Panel D: Equivalent numbers of MCF7 and pII cells (10^4^) were seeded into 12-well plates and grown over 6 days. Cells were harvested from triplicate wells on the days indicated and growth determined by cell counting. Differences at days 4, 5 and 6 were significant with *p*≤0.0001.

MCF7 cells typically grow as closely packed colonies forming sheet–like monolayer structures upon achieving confluency. In contrast, pII cells showed distinct morphological differences under the same growth conditions, as illustrated in Fig. 1, assuming a generally smaller more variable elongated and spindle or sometimes round shape, growing near to each other but forming loosely associated colonies of cells even at high density. Figure 1 panel C shows a quantitative analysis of the size/shape parameters of the two cell lines using ImageJ software. Time lapse photography showed that pII cells assumed a roundish appearance prior to cell division and then separated from each other whereas MCF7 cells remained together (data not shown). pII exhibited about 50% faster growth rates than MCF7 (Fig. 1D).

### Motility

**Figure 2:**
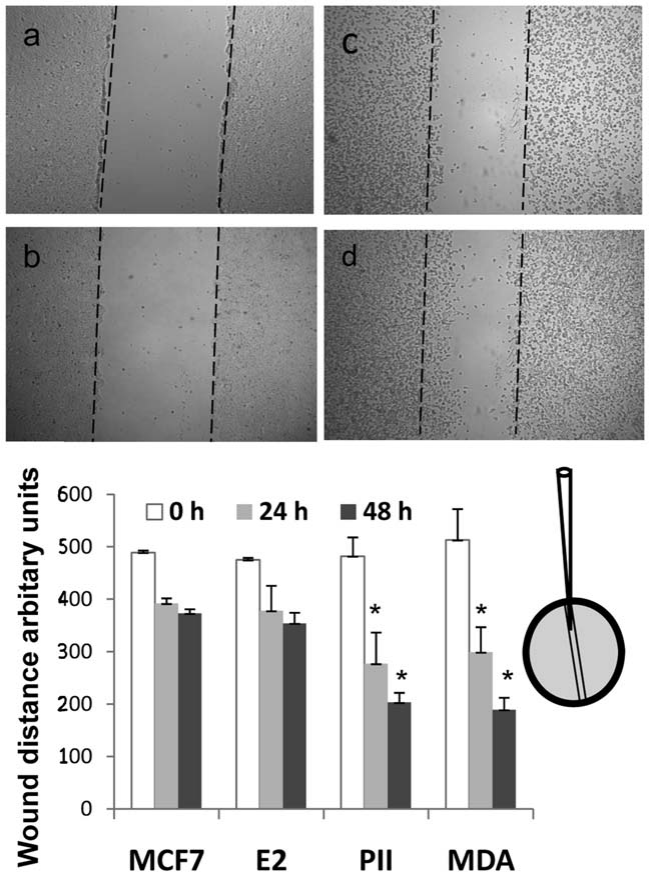
Motility assay of MCF7, pII and MDA231. Cells were plated into individual wells of a 6 well microwell dish and allowed to grow to ∼80% confluency. A scratch was made across the centre of the cell monolayer with a yellow eppendorf pipette tip (as shown in cartoon) creating a parallel space. Damaged cells were removed by carefully aspirating off the medium and rinsing with PBS before addition of fresh medium containing 2% serum. Upper panels show comparative microscopic view of MCF7 (a–b) and pII (c–d) cells in the scratched area at 0 time (a, c) and after 24 h [b, d] for a typical experiment. Chart shows the combined quantitated results from 3 independent experiments for the cell lines indicated. Histobars represent the mean [± standard deviation] width of the original scratch space at 0, 24 and 48 h. Wound closure indicates movement of cells into the scratch. *Significantly different from MCF7 cells at respective times with *p*≤0.006.

A pre-requisite for tumour cells to become invasive is loss of cell-cell adhesion to allow them to become more motile. We tested the ability of MCF7 and pII cells to move into a space created through an approximately 80% confluent monolayer as described in Methods. Although movement of some cells into the space could be seen after about 8 h accurate measurements could only be recorded from about 24 h. Observations made at longer periods do introduce additional complication that proliferation of cells that have moved into the scratch could contribute to the total number of cells seen within the scratch. The use of low serum in the medium is likely to minimise this effect, but at the same time it does reduce the movement of the cells to some extent, compared to normal serum conditions (data not shown). Another option sometimes employed is to use a mitotic inhibitor such as mitomycin C but this was not done in case this also affected motility. These considerations aside, we noted a significant reduction in the width of the scratch for pII as compared to both MCF7 and E2 cells indicating that pII cells had moved laterally into the space to a far greater extent. The change in the width of the scratched region from 0 to 24 and 48 h is displayed in Fig. 2. The ER -ve MDA231 cells behaved similarly to the pII, moving into the scratched space.

### Distribution of the actin cytoskeleton

**Figure 3:**
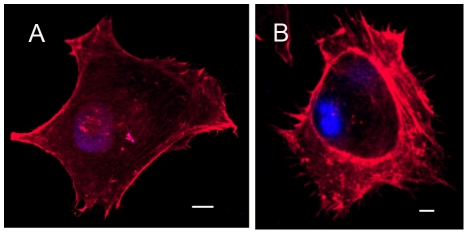
Confocal laser scanning microscope analysis of individual MCF7 (A) and pII (B) cells showing differences in both intensity and arrangement of F actin filaments visualised by phalloidin staining as described in Methods. Microspikes and lamellipodia-like structures are visible in the pII cells. Both photographs were enhanced equally by auto-contrast in Adobe Photoshop. Scale bar indicates 10 µm.

Following staining with phalloidin, pII cells exhibited an extensive and complex array of F-actin-rich structures that may represent lamellipodia and microspikes at the cell periphery, a phenomenon generally referred to as membrane ruffling. On the other hand, MCF7 cells showed a more regular distribution of F-actin filaments throughout the cells with no extensive membrane ruffling (Fig. 3).

### Cell invasion

**Figure 4:**
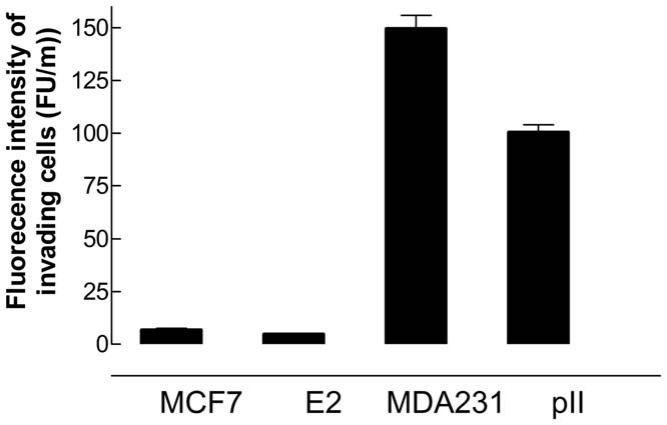
Invasion assay of MCF7, E2, pII and MDA231 cells through simulated ECM protein components. Cells were plated into the upper chambers of the cell invasion plate and incubated for 48 h prior to measurement of the fluorescence intensity of the invading cells in the bottom chambers. MCF7 and E2 cells were considered non-invasive (<20 FU/m), whereas pII and MDA231 cells progressively invaded the BME (>100 FU/m). Histobars represent the mean ± SEM of at least 3 independent determinations of the fluorescent intensity of the invading cells in the bottom chamber. This experiment was performed three times with similar outcome. pII and MDA231 significantly different from MCF7 with *p*<0.0001.

**Figure 5:**
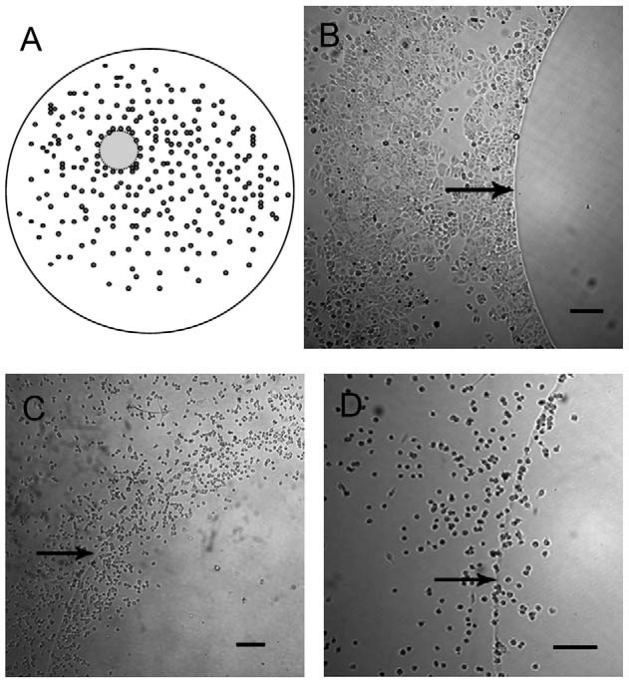
Agarose spot assay. Panel A is a cartoon to illustrate agarose spot inside well with cells added some of which settle around the periphery. MCF7 (B) and pII (C, D) cells were plated into individual wells of a 12 well dish containing an agarose spot, and photographed after 24 h of incubation. MCF7 cannot penetrate even at high density whereas pII do so even at very low density (D). Arrows delineate the periphery of the agarose spot beneath. Scale bar represents 400 µm. This experiment was repeated at least ten times with identical outcome. The numbers of pII cells entering the agarose varied with density of cells settling at the periphery, and time.

Invasion of cancer cells through the basement membrane into the extracellular matrix (ECM) is an essential step in cancer metastasis. In this assay, both pII and MDA231 demonstrated the ability to pass through the BME, indicated by fluorescent signals from the invading cell population in the bottom chamber of the cell invasion plate. In comparison, the fluorescent signal from the medium in the lower chamber for MCF7 was very low indicating that these cells were limited in their ability to penetrate through the BME (Fig. 4). Polysaccharides also constitute a major structural part of the ECM. Therefore we assessed the ability of the cells to invade through this component using the agarose spot assay described by Wiggins and Rappoport [41]. MCF7 cells were seen to accumulate at the agarose junction but did not enter the agarose. Even after 72 h when the cells were extremely confluent, they simply piled up against the agarose and were clearly unable to enter it. In sharp contrast, the pII cells were seen to penetrate into the agarose spot and accumulate in increasing numbers as time progressed, as illustrated in Fig. 5. These movements were observed irrespective of whether there was high or low initial density of pII cells at the agarose periphery.

### Transcriptome profiling

**Figure 6:**
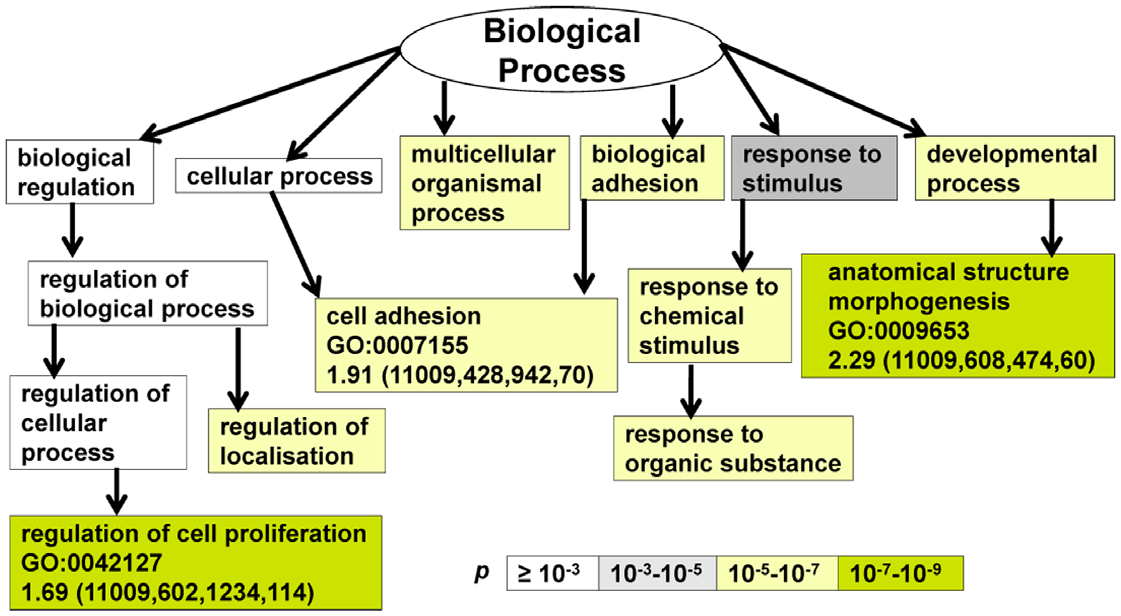
Biological processes affected by ER silencing. GO terms most significantly affected or disrupted secondary to ER silencing. Each GO term is boxed in a rectangle and shaded according to the *p* value as indicated. Enrichment (N, B, n, b), where indicated is defined as (b/n)/(B/N), where N - is the total number of genes, B - is total number of genes associated with a specific GO term (‘target’ set and ‘background set’), n - is number of genes in the ‘target set’, b - is number of genes in the ‘target set’ associated with a specific GO term.

**Figure 7:**
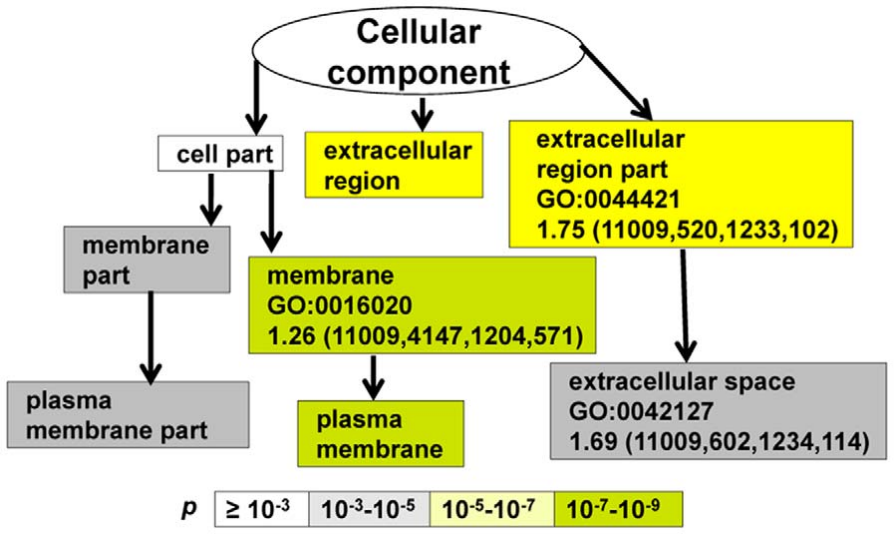
Cellular components affected by ER silencing. See legend to Figure 6.

EMT and related therapeutic resistance are complex biological processes, likely to involve participation of many genes, and may be addressed by the use of an unbiased method such as microarray. To that end, we utilized the Affymetrix GeneChips in an attempt to decipher transcriptomic alteration secondary to ER silencing. GO analysis has emerged as a powerful tool aimed at organizing gene expression data into a meaningful output associated with biological processes [42]. Using Anova *p* value set at <10^−6^ as the representative for the ranked and differentially expressed genes between MCF-7 and pII cells, nine GO terms emerged as the most significant biological processes disregulated upon ER silencing (Fig. 6). Consistent with higher growth rate and the altered morphology seen in pII cells, anatomical structure morphogenesis (GO:0009653), regulation of cell proliferation (GO:0042127) and cellular adhesion (GO:0022610 and GO:0007155), were among the most significant GO terms altered after silencing ER. Moreover, Fig. 7 shows that the majority of deregulated genes coded for plasma membrane associated or extra-cellular proteins (GO:0005886 and GO:0005576 respectively). The complete gene lists and analyses have been submitted (to geo@ncbi.nlm.nih.gov) and will be available on Gene Expression Omnibus website (http://www.ncbi.nlm.nih.gov/geo/query/acc.cgi?acc=GSE27473).

To look specifically at deregulated intracellular signalling pathways, we searched the entire unselected dataset using the Ingenuity Pathway Analysis (IPA) programme with specific reference to pathways associated with metastasis and invasion. We found that a number of changes had occurred in gene networks whose products have been implicated in cell to cell interactions and in cell motility. The majority of genes currently known to be involved in cellular interactions particularly those with functions associated with the extracellular space were up-regulated in pII cells (Fig. S1). The behaviour of genes involved in cell movement and adhesion was even more striking (Fig. S2), with all but two identified genes being up-regulated in pII. For more focussed analysis, a reductive process was used to restrict the data by imposing an arbitrary cut-off level of significance (*p*<10^−5^) and limiting consideration to sequences exhibiting geometric fold changes ≥3 and excluding those with no specific structural or functional assignment or were anonymous or had only chromosomal location. This left 2500 identifiable unique sequence differences between MCF7 and pII mRNA. Of these, approximately 1270 genes were comparatively up-regulated and the remainder down-regulated in pII. The ER regulated genes, PGR, CATHD and TFF1 and TFF3 as well as PRLR were predictably reduced. Amongst growth factors and their receptors, differences were observed between family members with the majority being higher in pII (Table 1). We also noted a clear distinction in expression of luminal and basal cell markers that have been identified as such in breast tumours [35] with the latter being consistently higher in pII (Table 2). We also looked for genes reported to be associated with invasion and metastasis (Table 3). Typical examples of pII up-regulated genes included vimentin and N-cadherin, fibronectin and tenascin C, WNT3, WNT5A and WNT5B, SMAD 3 and 4 and signalling molecules such as AKT and ZEB1, with concurrent decrease in E-cadherin, SMAD 1 and 6, keratin 7 and HOX genes. When we examined genes that have been designated as either epithelial or mesenchymal specific, almost without exception, all epithelial markers were down-regulated and mesenchymal markers up-regulated in pII (Table 4). A number of signalling molecules have been identified as key transcriptional drivers of EMT. Of these, SNAIL1 and 3, TWIST, ELK3 and TCF4 were unaltered whereas SNAIL2 (SLUG), ZEB 1 and ZEB2/SIP were higher in pII and TCF3/ E47/E12, and ELF3 were lower. The ER induced inhibitor of SNAIL1, MTA3, and GATA3, which inhibits breast cancer metastasis through the reversal of EMT [9], were also significantly lower.

**Table 1:** Peptide growth factor/receptor genes associated with endocrine resistance.

*Up regulated in pII*	*Down regulated in pII*
FGFR1, FGF1,5,13, EGFR, TGFα	FGFR2, 3, 4, FGF12, TGFβ3
TGFβ1, 2, TGFβR1, 2, 3	ERBB2, ERBB3, ERBB4
PDGFA, PDGFC, PDGFD	
VEGFA, VEGFC, HBEGF, CTGF	

**Table 2:** Luminal and basal markers.

***Luminal markers*** *	GATA3, XBP1, PRLR, MAPT, TIFF1, 3
***Basal Markers*** ^**+**^	EGFR, LYN, CAV1,2, MSN, ETS1, CD44,5
	AnnexinA1, WNT 5A, 5B, LAMC2

*downregulated in pII, +upregulated in pII.

**Table 3:** Genes associated with invasion and metastasis.

*Up-regulated in pII*	*Down-regulated in pII*
CDH12, FN1, TNC	CDH1,3, F11R, DSC2, DSP
WNT3, 5A,5B, JAG1, CAV1,2. SMAD 3,4	NOTCH3, SMAD 1,6
CALB2, ZEB1, EREG,TMEFF1, UGT8	MAN1A1, CLN8, TFF1
	KRT7, NEDD9
AKT2,3, CNK1, AXL, MSN, MAP1B	
PLEK2, MAP4	
VIM, CXCL1, TIMPs 1,2,3,4, SERPINE1	CXCR4, LY6E, BMP7
COX2, SAT, CALD1, RGS2, CAPG	HOX, HIST1H4

**Table 4:** Epithelial and mesenchymal markers.

Functional class	*Epithelial markers	^+^Mesenchymal markers
***Membrane glycosylated phosphoprotein***	MUC1	MUC15
***Matrix metalloproteinases***	MMPs9,15,17,24	MMPs1,14
***Cell cycle molecules***	Cyclin M1, M4	CyclinsA1, D2, E2
***Membrane proteins***	CD24, 42	CD44,59,68,73,99
***Cytoskeleton proteins***	Keratin 7,8,15,18, 19	VIM
***Adhesion molecules***	CDH 1,3,5,9,10,11,13,14,16,19	CDH4,8, 11, 12
	Protocadherins1,5,8–10,13,14,16,19	Pro-cadherin 7,18,20
	Annexin A9, Catenin, ICAM3	Annexin A1, ICAM1,2
	CLDN3,4,23 DSP, OCLN, TJP3	NCAM2, LOXL2
	GJB3, JPH3, JUP, SELL	Integrin α3, β1
		FN1,FLNC
***Collagen family***		Col 4A1,5A1,6A1,7A1
		12A1,13A1,4A5
		4A6,4A2,6A2,9A2
***Transcription factors***		GATA3, XBP1
***Heat shock protein***	HSP90AA1	HSPA1A
***Matrix associated protein***		SPARC
***Membrane receptors***	PRLR	
***Secretory molecules***	EDN1	
***Other***		PLAU, PLAUR, SMA

*Downregulated in pII, ^+^upregulated in pII.

### Gene expression analysis by realtime PCR

To validate the microarray data, relative levels of 12 randomly selected genes in the MCF7 and pII derived cDNA preparations were measured by realtime Taqman PCR amplification [data not shown]; in all cases the results correlated precisely. Amplifications were also performed with a further 8 genes; ER, keratin 19, E-cadherin, N-cadherin, fibronectin, vimentin, CD68 and PLAU for MCF7, pII and MDA231 cells (Fig. S3) confirming a clear division between epithelial and mesenchymal marker expression. Whereas MCF7 showed high expression of ER, keratin 19 and E-cadherin, the others were all greatly reduced. The reverse was observed for both pII and MDA231 cells.

### Timescale of EMT

**Figure 8:**
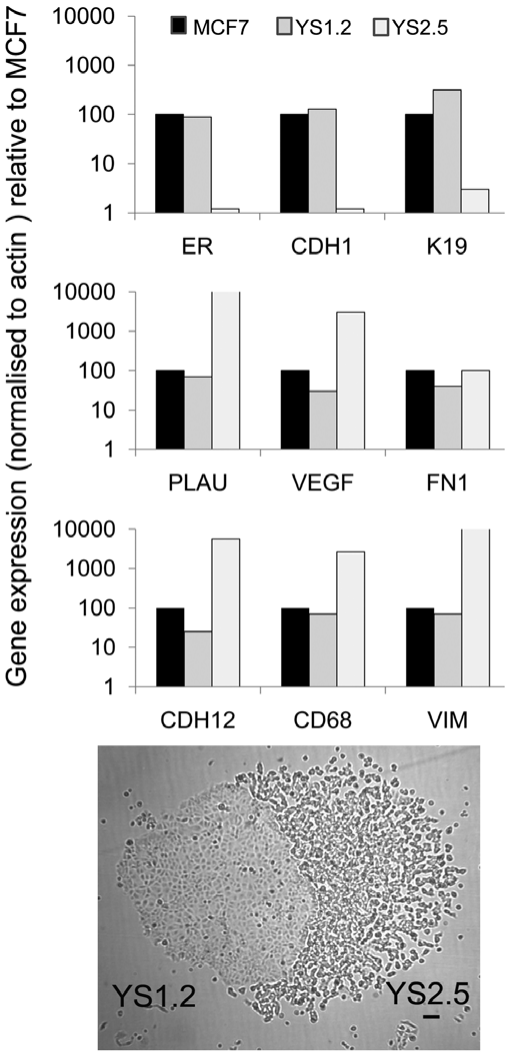
Realtime PCR analysis of epithelial and mesenchymal markers in MCF7, YS1.2 and YS2.5 cells. Extracted RNA was converted to cDNA and amplified using the Taqman procedure as described in Methods. Histograms represent means of duplicate determinations. Similar results were obtained in two separate experiments. Expression was normalized to the level found in MCF7 cells (arbitrarily fixed as 100), with β actin used as an internal control. Photomicrograph shows the morphology of the two original adjacent G418 resistant colonies from which YS1.2 and YS2.5 were cloned. Scale bar represents 100 µm.

The data presented here was obtained with pII cells that had been passaged in excess of 15 times following initial cloning. To determine how quickly after ER silencing the changes actually occurred, new transfections of MCF7 cells were performed using ER targeting shRNA cloned into pSingle-pTs vector and two adjacently growing transformants that displayed morphology similar to MCF7 and pII (Fig. 8 inset) were cloned and examined within 3 passages of selection. Expression of the gene set described previously was assessed by realtime PCR. The data in Fig. 8 shows that the MCF7-like clone had failed to down-regulate ER and exhibited a profile similar to the parental line whereas the one resembling pII exhibited almost complete loss of ER and developed the ‘EMT signature’ pattern.

## Discussion

In this study we show that ER+ve MCF7 breast cancer cells that have undergone enforced long term ER deprivation by siRNA silencing (pII cell line), and acquired an endocrine resistant state [30] with loss of estrogen regulated gene expression, display a new phenotype that has features distinctly reminiscent of mesenchymal cells. They have a faster growth rate, changed morphological appearance with loss of intercellular adhesion and cytoskeletal redistribution that promotes increased locomotive activity and interaction with the ECM. Elevated CAPG is consistent with loss of aggregation, invasion and motility observed with its down-regulation in ER-ve MDA231 cells [43]. Ingenuity Pathway Analysis of the Affymetrix screen data showed increased expression of many genes whose products are involved in cell motility and in cell-cell interactions located both within the cytoplasm and also extracellularly (Figs. S1 and S2) indicating the network of underlying molecular processes permitting pII cells to penetrate simulated components of the ECM similarly to MDA231 (described as a mesenchymal cell; [35]) that are not operative in MCF7. pII resemble basal-like metaplastic and claudin/occludin-low tumour subtypes, with loss of luminal cell markers (Table 2), that have the propensity for inter-cellular detachment by ‘cadherin switching’ [13], [44] from E-cadherin to N-cadherin, degradation of the basement membrane through the action of various metalloproteinases and molecules listed in Table 3 and establishment of connections with elevated collagen components of the ECM in order to crawl into and through it into the vasculature. Cancers undergoing EMT display cell intermediate filament status changes from a keratin rich network connected to adherens junctions and hemidesmosomes to a vimentin base connecting a focal adhesion [22]. Vimentin is expressed at sites of elongation suggesting its putative role in facilitating migration as demonstrated with pII cells. Its expression also correlates with cell migration, invasion and EMT induction in several breast cancer cell lines in association with other mesenchymal markers such as fibronectin and tenascin C [10], [31] both of which were highly up-regulated in pII, with accompanying loss of cytokeratins and the classic epithelial marker, MUC1. Over-expressed basal markers CAV 1 and 2 are key molecules for cell migration involved in plasma membrane dynamics and cellular trafficking [45].

Mitogenic signals through EGFR [46], IGFR [47] and FGFR1 [48] are implicated in tamoxifen resistance and correlated with invasiveness. We observed increased expression of EGFR, FGFR1 and TGFβR1, 2 and 3 as well as several growth factor ligands including TGFα, TGFβ and several FGFs, PDGFs and VEGFs as well as WNT genes (Table 1) that control signalling through the RAS, SRC, PI3K and SMAD pathways, which are all implicated in EMT. TGFβ1 binding to heterodimeric complexes of TGFβR1 and 2 can mediate phosphorylation of SMAD 2 and 3 which in conjunction with SMAD 4 translocates to promoters such as SP1 that regulates SNAIL1 [49], [50] and interacts with other transcription factors, co-activators and co-repressors to suppress epithelial genes and promote expression of mesenchymal proteins [51]. Non-SMAD signaling through activation of ERK MAPK, RHO GTPases, and PI3K/AKT has also been implicated in TGF-beta-induced EMT [52]. Annexin A1 can also increase cell scattering and SMAD 3/4 transcriptional reporter activity and actin reorganization, which facilitates an EMT-like switch [53].

pII have high expression of other transcriptional repressors of E-cadherin [17] that include SNAIL2, ZEB1 and ZEB2/SIP, which also indirectly promotes vimentin expression [54] as well as GOOSECOID and FOXC2 [55], [56]. Another up-regulated marker is CD44, a β-catenin/TCF/LEF-1 target and a cell surface adhesion molecule involved in cell-cell and cell-matrix interactions, in association with simultaneous CD24 down-regulation. Tumours with a CD44+/CD24- phenotype are highly invasive [57] with targeted reduction of CD44 markedly reducing malignant characteristics of tumours *in vivo*. MSN anchors CD44 and is included in a gene expression signature associated with cell motility and invasion [35].

MTA3 is a subunit of the Mi-2/NuRD histone deacetylase complex [58] which is a key component of an ER dependent pathway that is responsible for the suppression of SNAIL. The loss of ER function and hence decreased MTA3 in pII cells would lead to de-repression of SNAIL transcription, cadherin switching and progression towards EMT. Immunoreactivity for MTA3 also correlated with ER-ve status of breast tumours and lack of E-cadherin. Depletion of MTA3 does not however appear to affect SNAIL2 which in contrast to SNAIL was over-expressed in pII. It is therefore not clear to what extent the loss of MTA3 contributes to EMT in these cells. Interestingly, ligand-activated ER can mediate transcriptional repression of SNAIL2; an effect that could be reversed by ER silencing in MCF7 [59]. Moreover, Hugo and colleagues [60] conclude in a review on the role of EMT in cancer progression that there is more evidence to support a role for TWIST and SNAIL2 in the mesenchymal phenotype than SNAIL 1.

All of our data are consistent with the observation that loss of ER in MCF7 cells drives them towards EMT. The pII cells have acquired characteristics very similar to those exhibited by the MDA231 cells that were originally isolated from a *de novo* ER –ve tumour. The key players that have been identified as either promoted or suppressed during EMT are frequently similar to those described for endocrine resistant cells. It is also clear that a large number of diverse signalling pathways are involved in both processes. Their relative importance remains to be determined, as does the temporal sequence of events. Our initial notion that loss of ER would lead to a gradual adaptation into an estrogen independent phenotype, as appears to be the case with most endocrine resistant cell models described in the literature, led us to examine these cells only after continuous propagation over many cell divisions to ensure their stability. That both endocrine independence and EMT can in fact occur quite rapidly following ER silencing is indicated very clearly by the preliminary analysis of the more recent transfectants YS1.2 and YS2.5 that shows acquisition of our ‘EMT signature’ is actually acquired very soon after stable selection.

In summary our data point to the conversion of MCF7 cells into an endocrine resistant state triggered by the functional loss of ER, which directly and swiftly initiates a complex series of processes that lead to EMT, resulting in an aggressive re-orientated and highly metastatic cell. Recognition of the link between these processes is already leading to exploration of newer intervention strategies [61] to address the clinical issue of endocrine resistance. A recent review [62] discusses the growing, often circumstantial evidence that breast cancer metastasis may be regarded as a specific form of EMT.

## Supporting Information

Figure S1 **A molecular interaction node depicted by Ingenuity Pathway Analysis centered on molecules involved in cell to cell interaction and subdivided into cellular component/location.** The nodal relationships are shown by solid lines indicating direct known interactions and dashed lines for indirect interactions. Additionally, the shape of each node indicates the molecular class. Red nodes indicate overexpression in pII cells and green nodes underexpressed transcripts, with un-coloured nodes as unaltered expression.
(TIF)

Figure S2 **A molecular interaction node depicted by Ingenuity Pathway Analysis centered on molecules involved in cellular motility and subdivided into cellular component/location.** The nodal relationships are shown by solid lines indicating direct known interactions and dashed lines for indirect interactions. Additionally, the shape of each node indicates the molecular class. Red nodes indicate overexpression in pII cells and green nodes underexpressed transcripts, with uncoloured nodes as unaltered expression.
(TIF)

Figure S3 **Realtime RT-PCR analysis of epithelial [ER, CDH1, K19] and mesenchymal (PLAU, VIM, CDH12, CD68, FN1 and VEGF) markers in MCF7, pII and MDA231 cells.** Extracted RNA was converted to cDNA and amplified using Taqman procedures as described in Methods. Expression was normalized to the ΔΔCt value for MCF-7 cells, with β actin used as an internal control. Data is expressed as a fold change from MCF7 which was arbitrarily fixed at 100. Histobars represent means of duplicate determinations. Similar results were observed in two separate experiments.
(TIF)
