# Supplementary figures and images for: Estrogen Receptor Silencing Induces Epithelial to Mesenchymal Transition in Human Breast Cancer Cells
Source: PLoS One. 2011 Jun 21;6(6):e20610. doi: 10.1371/journal.pone.0020610 (PMC3119661; doi:10.1371/journal.pone.0020610)

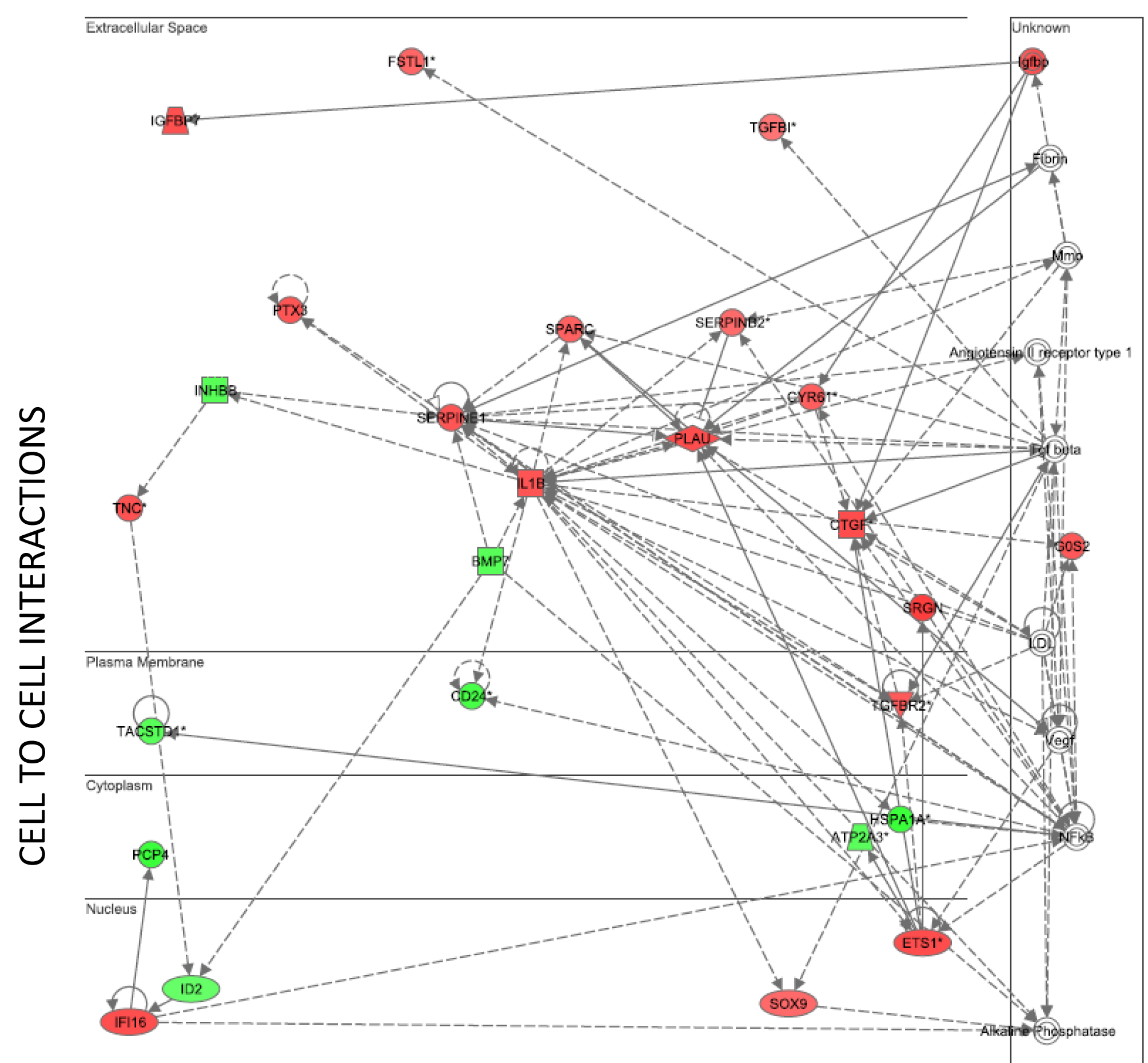

Supplement: Figure S1 — A molecular interaction node depicted by Ingenuity Pathway Analysis centered on molecules involved in cell to cell interaction and subdivided into cellular component/location. The nodal relationships are shown by solid lines indicating direct known interactions and dashed lines for indirect interactions. Additionally, the shape of each node indicates the molecular class. Red nodes indicate overexpression in pII cells and green nodes underexpressed transcripts, with un-coloured nodes as unaltered expression. (TIF) [file pone.0020610.s001.tif]

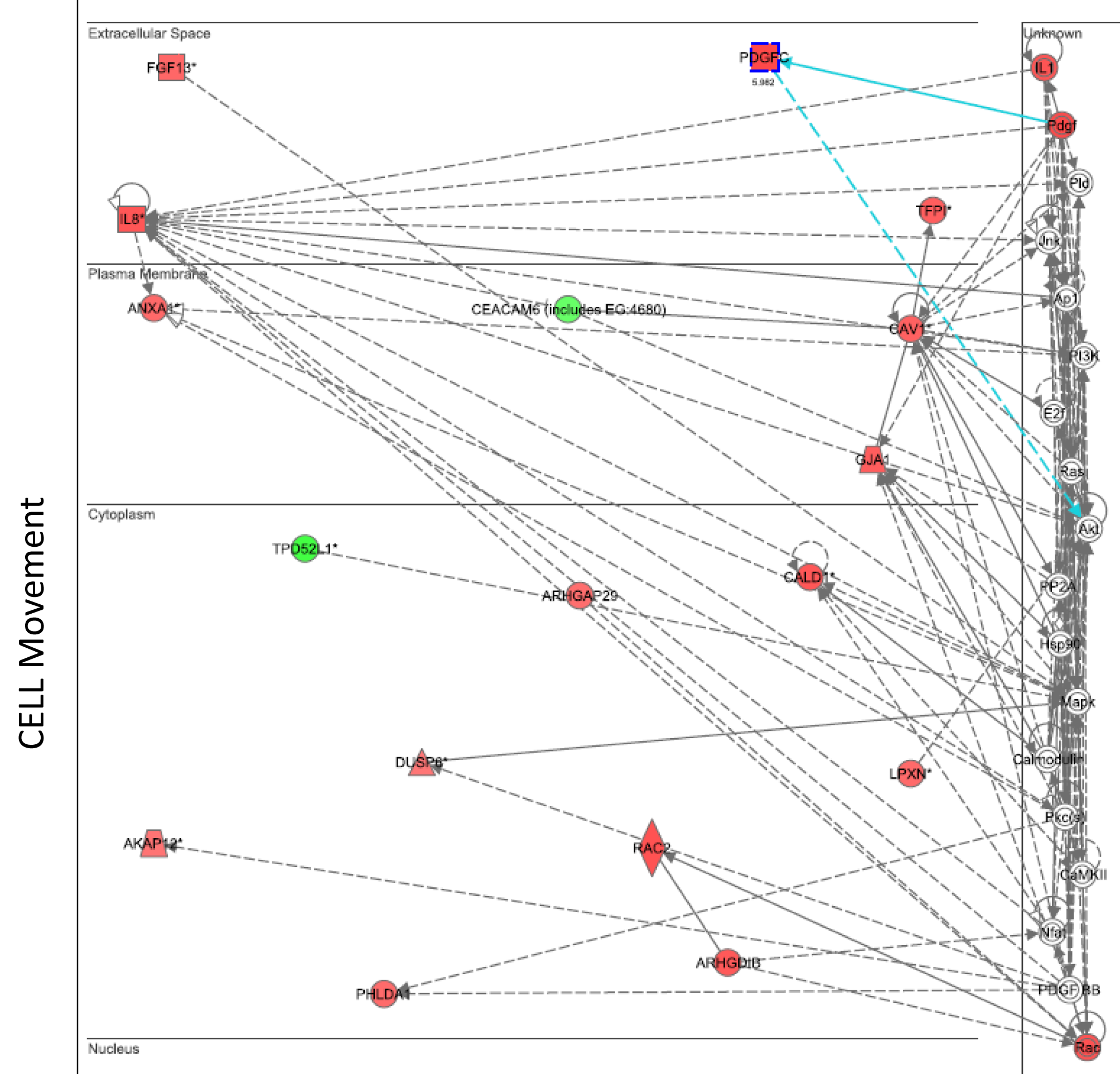

Supplement: Figure S2 — A molecular interaction node depicted by Ingenuity Pathway Analysis centered on molecules involved in cellular motility and subdivided into cellular component/location. The nodal relationships are shown by solid lines indicating direct known interactions and dashed lines for indirect interactions. Additionally, the shape of each node indicates the molecular class. Red nodes indicate overexpression in pII cells and green nodes underexpressed transcripts, with uncoloured nodes as unaltered expression. (TIF) [file pone.0020610.s002.tif]

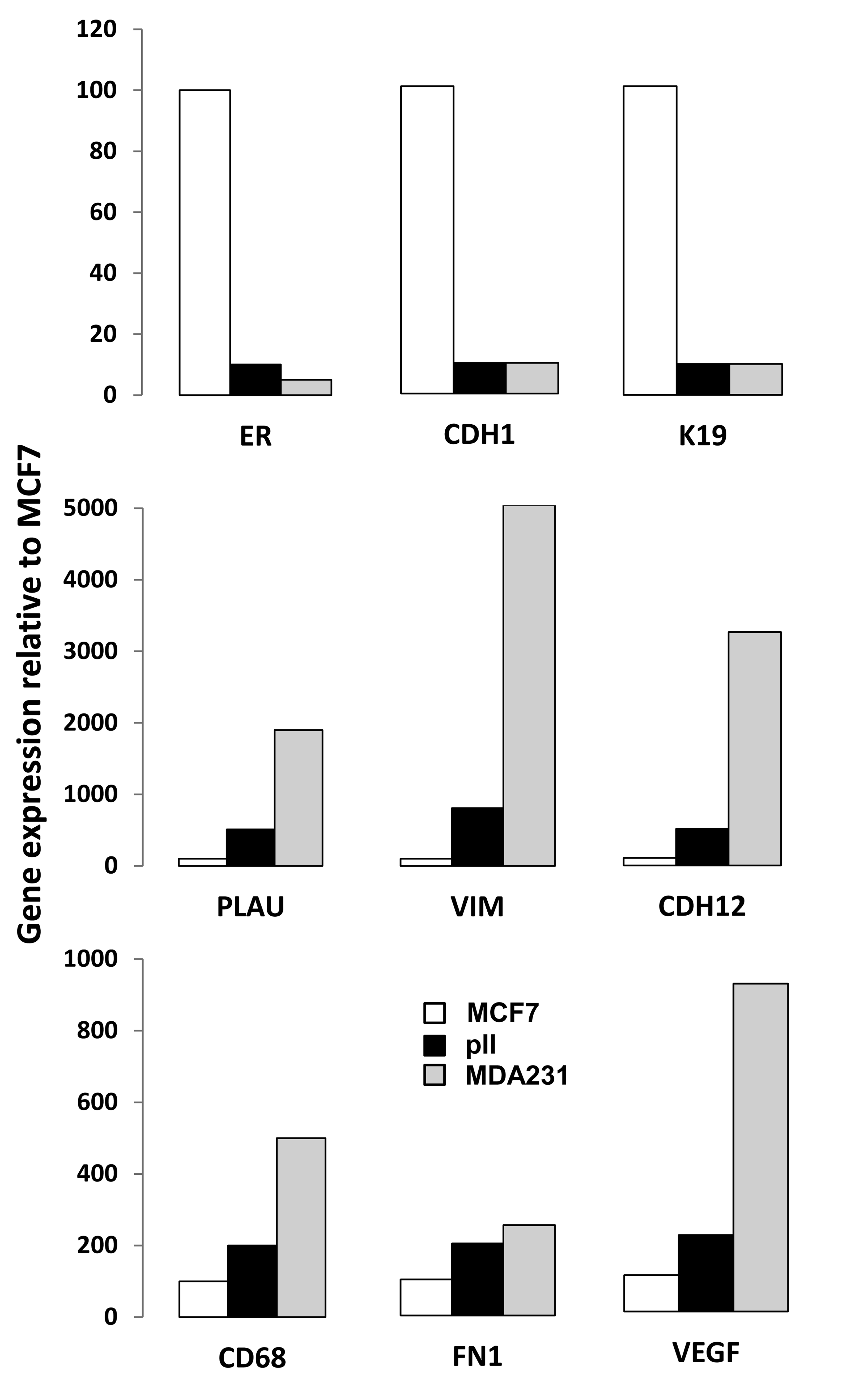

Supplement: Figure S3 — Realtime RT-PCR analysis of epithelial [ER, CDH1, K19] and mesenchymal (PLAU, VIM, CDH12, CD68, FN1 and VEGF) markers in MCF7, pII and MDA231 cells. Extracted RNA was converted to cDNA and amplified using Taqman procedures as described in Methods. Expression was normalized to the ΔΔCt value for MCF-7 cells, with β actin used as an internal control. Data is expressed as a fold change from MCF7 which was arbitrarily fixed at 100. Histobars represent means of duplicate determinations. Similar results were observed in two separate experiments. (TIF) [file pone.0020610.s003.tif]
